# Chromatic discrimination in fixed saturation levels from trichromats and subjects with congenital color vision deficiency

**DOI:** 10.1038/s41598-022-09531-z

**Published:** 2022-04-04

**Authors:** Yuzo Igarashi, Luiza Karina Gonçalves Meireles, Felipe André Costa Brito, Leonardo Ramos Nicolau da Costa, Joyce dos Santos Freitas, Leticia Miquilini, Luiz Claudio Portnoi Baran, Leonardo Dutra Henriques, Einat Hauzman, Daniela Maria Oliveira Bonci, Marcelo Fernandes Costa, Dora Fix Ventura, Paulo Roney Kilpp Goulart, Givago Silva Souza

**Affiliations:** 1 Núcleo de Teoria e Pesquisa do Comportamento, Universidade Federal do Pará, Av. Generalissimo Deodoro 92, Umarizal, Belém, 66055-240 Brazil; 2 Instituto de Ciências Biológicas, Universidade Federal do Pará, Belém, Brazil; 3 Núcleo de Medicina Tropical, Universidade Federal do Pará, Belém, Brazil; 4 Departamento de Psicologia Experimental, Instituto de Psicologia, Universidade de São Paulo, São Paulo, Brazil

**Keywords:** Visual system, Colour vision

## Abstract

Color vision tests use estimative of threshold color discrimination or number of correct responses to evaluate performance in chromatic discrimination tasks. Both approaches have advantages and disadvantages. In the present investigation, we compared the number of errors during color discrimination task in normal trichromats and participants with color vision deficiency (CVD) using pseudoisochromatic stimuli at fixed saturation levels. We recruited 28 normal trichromats and eight participants with CVD. Cambridge Color Test was used to categorize their color vision phenotype, and those with a phenotype suggestive of color vision deficiency had their L- and M-opsin genes genotyped. Pseudoisochromatic stimuli were shown with target chromaticity in 20 vectors radiating from the background chromaticity and saturation of 0.06, 0.04, 0.03, 0.02, 0.01, and 0.005 u’v' units. Each stimulus condition appeared in four trials. The number of errors for each stimulus condition was considered an indicator of the participant's performance. At high chromatic saturation, there were fewer errors from both phenotypes. The errors of the normal trichromats had no systematic variation for high saturated stimuli, but below 0.02 u’v' units, there was a discrete prevalence of tritan errors. For participants with CVD, the errors happened mainly in red-green chromatic vectors. A three-way ANOVA showed that all factors (color vision phenotype, stimulus saturation, and chromatic vector) had statistically significant effects on the number of errors and that stimulus saturation was the most important main effect. ROC analysis indicated that the performance of the fixed saturation levels to identify CVD was better between 0.02 and 0.06 u’v’ units reaching 100%, while saturation of 0.01 and 0.005 u’v’ units decreased the accuracy of the screening of the test. We concluded that the color discrimination task using high saturated stimuli separated normal trichromats and participants with red-green color vision deficiencies with high performance, which can be considered a promising method for new color vision tests based in frequency of errors.

## Introduction

Different research groups have been developing computerized tests for a psychophysical color vision evaluation, such as the Cambridge Colour Test (CCT) and the Colour Assessment Diagnosis (CAD), applying different testing strategies to quantify congenital and acquired color vision deficiencies^1–4^. CCT and CAD are color vision tests that implemented methods to estimate color discrimination thresholds along different chromatic vectors in chromaticity color spaces^5–7^. Both tests apply a forced-choice method in the psychophysical task to feed a staircase that controls the chromatic distance between the target and background chromaticity coordinates. The staircase procedure can be very time-consuming since it requires several reversals or trials to complete. Nevertheless, the completion of the staircase procedure results in the estimation of color discrimination thresholds at different chromatic vectors, which can be taken as indicators of the color vision performance for the perceptual task. Fast approaches such as the trivector test from CCT, which the chromatic discrimination task occurs only in the chromatic axis of the color confusion lines. CCT trivector protocol has been used to shorten the test duration. The disadvantage of these short approaches is the reduction in the number of chromatic axes to be tested, especially in case of acquired color vision deficiency (CVD) evaluation.

While CCT and CAD were designed to quantify the color discrimination thresholds of a subject, another interesting approach to evaluate color vision was suggested by Shin et al.^8^. The authors used a computerized test showing pseudoisochromatic stimuli with suprathreshold chromatic contrast for normal trichromats. The observers were asked to identify the shape of a target or its absence. They used eighty combinations of lightness, saturation, and hue and recorded the number of errors during the test to measure the individual color vision performance. In that investigation, the chromatic discrimination was applied to compare diabetic patients with healthy trichromats. Instead of estimating thresholds, the authors investigated the frequency of errors in target identification, and they observed that the performance of the diabetic patients was worse than healthy trichromats and that the color vision loss was consistent with a diffuse acquired color vision loss.

The computerized proposal of Shin et al.^8^, named SNU color test, is similar to many printed color vision tests. In the same test, they used stimulus with different combination of lightness (3 values), chromaticity (16 values) and saturation (6 values). For a given saturation level, the chromatic vector size was equal distance from the central white point in the HSV color space. The performance of the participant was based in the number of errors of the observer.

In the present study, we intended to explore this approach using more controlled stimulus conditions (same lightness, saturation, chromaticity and target shape), and testing participants with congenital CVD in fixed saturation levels. We consider that these additions would improve the comprehension of this approach in order to design other color vision tests based in the estimating of frequency of errors. We think that the Shin et al.^8^ approach could be applied using fixed saturation levels and evaluated the performance of each saturation level to identify the presence of CVD. The comparison of the performance of different fixed saturation stimulus levels to screen the color vision deficiency can be useful to design fast color vision tests using only the smaller fixed saturation level with a desired criterion of screening. In addition, the use of C-gap as target enables the execution of a simple positional localization task which is more related to retinal and primary visual cortex processing than the target form discrimination that is processed in higher visual areas^9^. Testing participants with congenital CVD is very important to test the specificity of the test to identify predictable error during testing condition using colors that confuse participants with color vision deficiencies.

## Material and methods

### Ethics

An informed written consent was obtained from all the participants after being informed about the objectives and all steps of the investigation. This study was carried out following the recommendations of National Health Resolutions, the National System of Research Ethics of Brazil, conducted under the Declaration of Helsinki 1964 and posterior updates. Furthermore, we received the approval of the Ethical Committee for Research in Humans of the Núcleo de Medicina Tropical, Universidade Federal do Pará (report # 2.274.540), Brazil.

### Subjects

Our sample comprised 28 normal trichromats (14 males, 14 females, 24.2 years old ± 4.3) and eight participants with congenital red-green CVD (7 males, 1 female, 22.5 years old ± 3.2). All participants had no history of systemic or neurological disease that could impair any visual function and no medication affecting the central nervous system. In addition, all had normal or best-corrected visual acuity to 20/20.

### Characterization of the color vision

First, all participants were evaluated using CCT (Cambridge Research System, Rochester, CRS, United Kingdom) in the 20 chromatic vectors protocol to estimate color discrimination thresholds. For all subjects whose ellipse’s major axis was lengthier than the upper limit of the normative data, we performed the genotyping procedure to identify the genomic presence of the M- and the L-opsin genes, based on the method proposed by Neitz and Neitz^10^ to confirm that the CVD has a congenital background. The method amplifies a 300 bp fragment containing the exon 5 of both visual opsins genes, using polymerase chain reactions (PCR) and the primers 5’TCCAACCCCCGACTCACTATC and 5’ACGGTATTTTGATGTGGATCTGCT, followed by a restriction endonuclease reaction. DNA samples were collected and extracted from a buccal brush and purified using the Gentra Puregene Buccal Cell Kit (Gentra Systems, Inc., Minneapolis, MN, USA), following the manufacturer’s protocol. PCRs were carried out using High Fidelity Platinum^®^ Taq Polymerase, 10 × High Fidelity Buffer, MgCl2, 10 mM GeneAmp dNTPs (Applied Biosystems, Inc., Foster City, USA), and 20 mM primers in 50 µl reactions, and PCR conditions were described elsewhere^11^. The amplified fragments were incubated with the restriction endonuclease Rsa I (Invitrogen, Carlsbad, USA), which recognizes and cleaves the exon 5 of the L-opsin gene, resulting in two smaller fragments with about 100 bp and 200 bp, but do not cleave the M-opsin gene, resulting in a single ~ 300 bp fragment. The resulting PCR products were visualized in 1.0% agarose gel electrophoresis. Trichromat individuals, with both L and M genes, were identified by the presence of three bands (a ~ 300 bp band from the uncleaved exon 5 of the M-opsin and two smaller bands of the cleaved exon 5 of the L-opsin). This method identifies the absence of M-opsin or L-opsin gene, it cannot separate when the individual is dichromat or anomalous trichromat. Deuteranope and deuteranomalous individuals (no M-opsin gene) display the two smaller bands, while protanope and protanomalous individuals, with no L-opsin gene, display only one ~ 300 bp band. The DNA sample from a known trichromat was used as a control.

### Color discrimination evaluation in fixed saturation level

**Figure 1:**
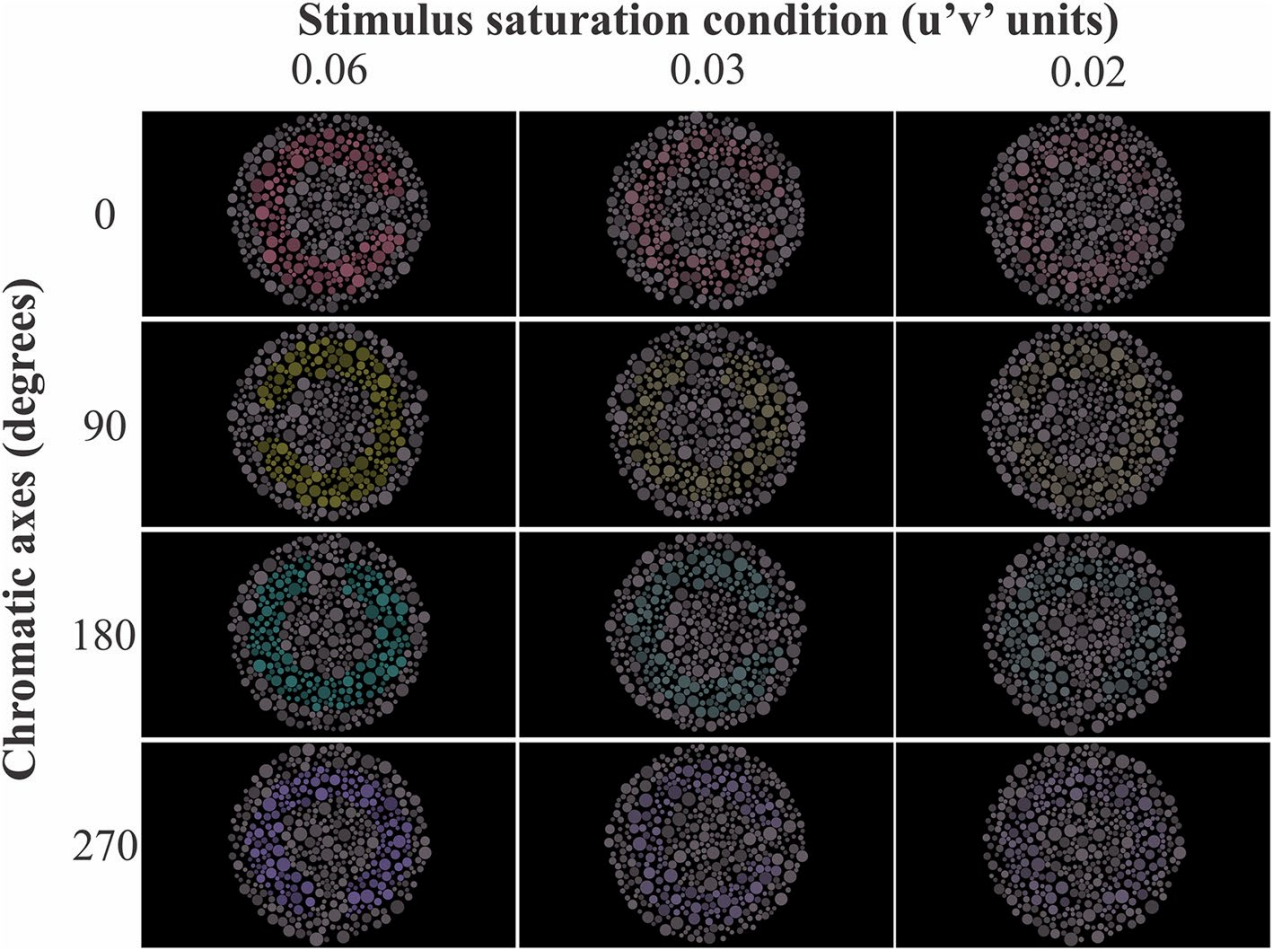
Examples of pseudoisochromatic stimuli for three chromatic saturations (0.06, 0.02, and 0.005) and four chromatic axes (0°, 90°, 180°, 270°).

We programmed a test for color discrimination evaluation in fixed saturation levels in MATLAB language environment (Mathworks, MA, USA), using a graphic library of the ViSaGe system (CRS toolbox for MATLAB, Cambridge Research System, Rochester, UK). The stimuli were 6.5° circular pseudoisochromatic mosaic composed of 450 circles ranging 10 radii from 0.07° to 0.23° of visual angle. The luminance of the circles was randomly defined with one of six equally spaced values between 8 and 18 cd/cm^2^. Each mosaic had a C-shaped subset of circles in the mosaic center whose chromaticity differed from the remaining mosaic, forming a target-background arrangement (Fig. 1). The spatial dimensions of the target were: outer diameter of 4.4°, inner diameter was 2.2°, and the C-gap was 1° of visual angle. The background chromaticity was u’ = 0.1987 and v’ = 0.48.

We used CIE 1976 color space to define the stimuli with parameters similar to those used in CCT. During the test, target chromaticity varied along 20 vectors in the CIE 1976 color diagram, radially distributed around the background chromaticity coordinate, and equally spaced in 18°. Six saturation conditions (chromatic vector) were tested (0.06, 0.04, 0.03, 0.02, 0.01, and 0.005 u’v’ units). The saturation values represent the distances from the coordinate of background chromaticity. The target was shown during 2 s and followed by a dark screen. The subject’s task was to inform the orientation of the C-gap (right, left, top, bottom) by pressing the corresponding button in a 4-button box (CRS). Each target chromaticity was shown in 4 trials, totaling 480 trials (6 fixed saturation level × 20 chromatic axes × 4 trials). The target orientation and the saturation condition of the target were randomly chosen. The indicator of the participant’s performance was the number of errors. The greater the number of errors, the worse the performance.

### Data analysis

The chromatic discrimination across stimuli conditions was estimated by the number of errors. A three-way ANOVA with Geisser-Greenhouse correction was conducted to compare the main effects of color vision phenotype (two levels, trichromacy and congenital color vision deficiency), chromatic saturation (six levels), chromatic vector (20 levels), and their interaction in the number of errors. Partial eta squared was calculated the effect size of the main effects. We considered a confidence level of 95% for all the statistical procedures.

We applied a Receiver-Operating Characteristic (ROC) analysis to evaluate the accuracy of each fixed saturation level to screen the presence of color vision deficiency. For that, we considered the cumulative distribution of correct responses from normal trichromats as “correct responses” and the cumulative distribution of the correct responses from the color vision deficient participants was considered the “false alarms”. The area under the curve (AUC) was considered the quantification of the accuracy of the classifier to screen the presence of color vision deficiency. A MATLAB (Mathworks, Natick, CA) routine was written to proceed with the statistics.

## Results

### Color vision characterization

**Figure 2:**
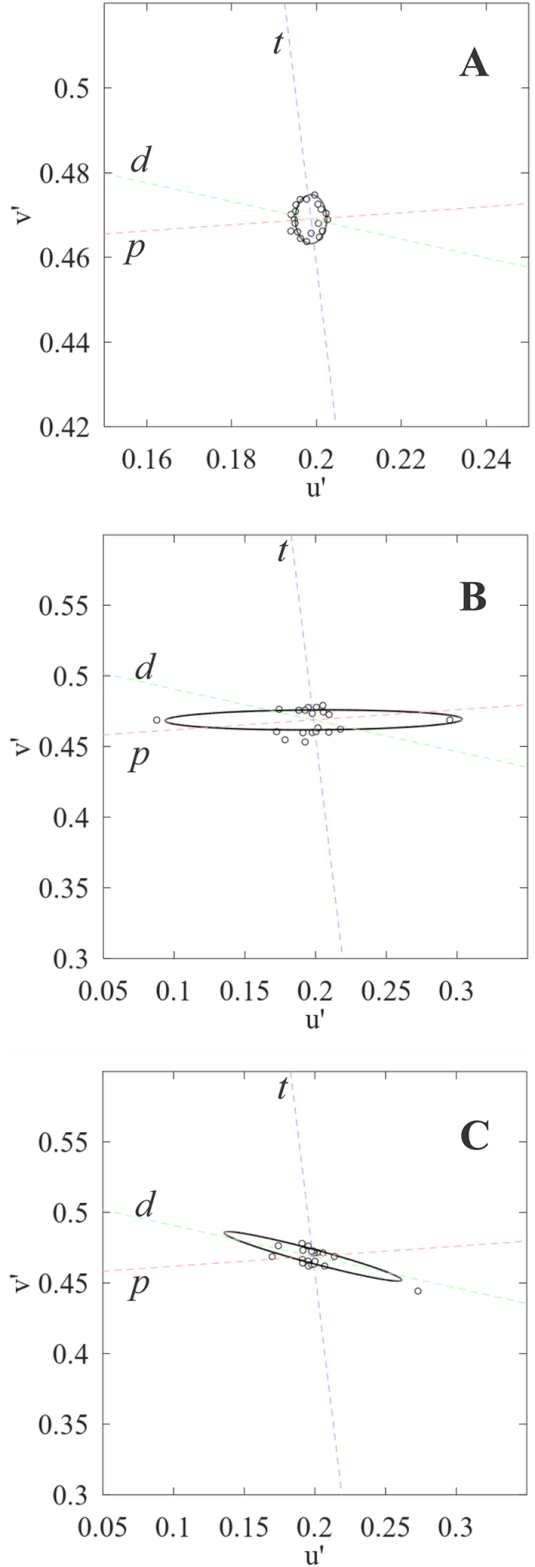
Chromatic discrimination ellipses from a normal trichromat **(A)**, and participants with CVD having protan defect **(B)** and deutan defect **(C)**. Circles represent chromatic discrimination thresholds at 20 chromatic axes. Dashed lines represent protan (*p*), deutan (*d*), and tritan (*t*) confusion lines.

Figure 2 shows the color discrimination ellipses from representative trichromat and participants with CVD. All participants with poor chromatic discrimination in the CCT evaluation had their color vision genotyped to confirm the congenital feature of the CVD. Table 1 shows the color vision characterization for the participants with CVD of the present study, with genotyping and CCT results for each subject, expressed by ellipse major axis length and angle of rotation of the ellipse. All of eight participants with CVD had agreement between the suggestive color vision loss in CCT and the absence of M-cone and L-cone genes.

**Table 1:** Results of the color vision characterization of the dichromats/anomalous trichromat participants.

Participant	CCT result			Genotype
	Ellipse major axis length (u’v’ units × 10^–4^)	Angle of rotation of the ellipse (degrees)	Suggestive color vision loss	
P1	1051	0.7	Protan	No L-opsin gene
P2	976	0.3	Protan	No L-opsin gene
D1	651	162	Deutan	No M-opsin gene
D2	788	165	Deutan	No M-opsin gene
D3	512	160	Deutan	No M-opsin gene
D4	516	168	Deutan	No M-opsin gene
D5	655	164	Deutan	No M-opsin gene
D6	572	163	Deutan	No M-opsin gene

### Chromatic discrimination in fixed saturation levels

**Figure 3:**
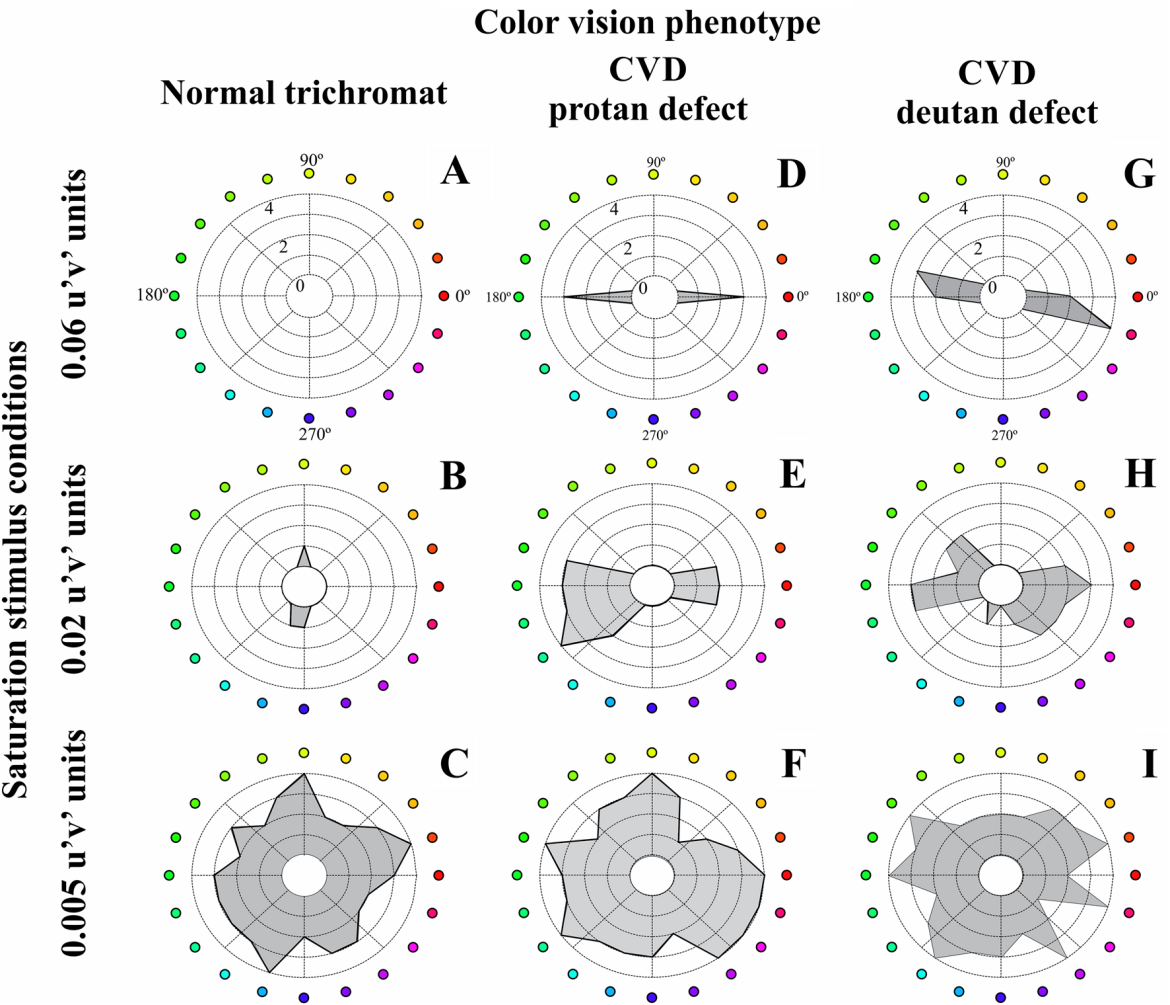
Polar representation of the chromatic discrimination in fixed saturation levels as proposed at the present study for a normal trichromat **(A–C)**, a participant with CVD and protan defect **(D–F)**, a participant with CVD and deutan defect **(G–I)** at three stimulus saturations (0.06, 0.02, 0.005 u’v’ units). In the polar plot, the radius of the circle represents the number of errors (from 0 to 4) during the chromatic discrimination task. The angle represents the chromatic axes. Shaded areas represent the errors in the different chromatic vectors.

**Figure 4:**
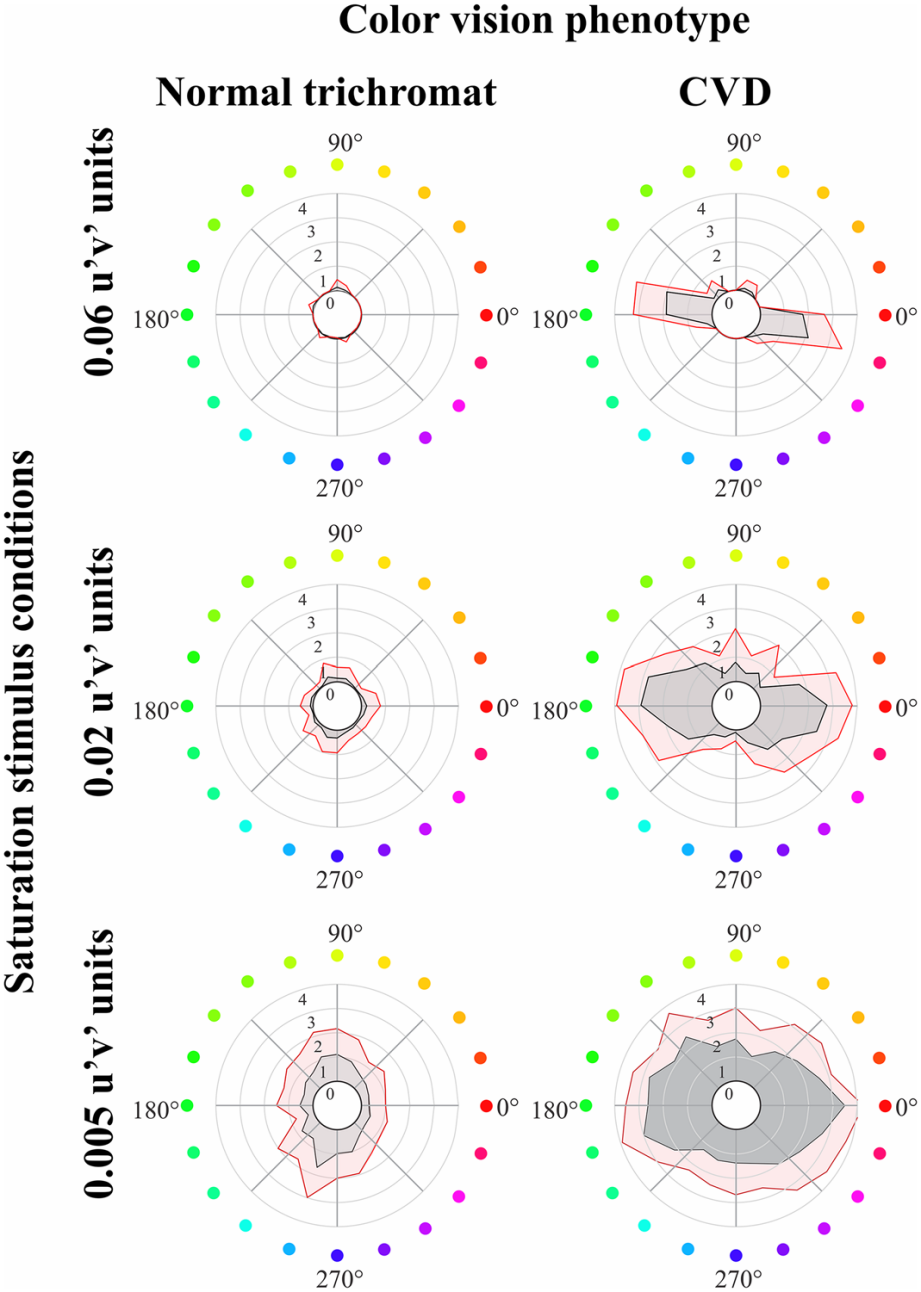
Mean values and dispersion for the polar representation of the chromatic discrimination in fixed saturation levels. **(A)** Normal trichromats. **(B)** Participants with CVD. Shaded gray areas represent the mean errors in the different chromatic vectors, while shaded red areas represent the mean errors plus 1 standard deviation in the different chromatic vectors.

Figure 3 shows the results of the chromatic discrimination in fixed saturation levels of representative participants from trichromat group, participant with CVD group having protan defects, and having deutan defects for the different chromatic saturation conditions. The angulation of the errors of participants with CVD were close from the color confusion lines protan or deutan. Figure 4 represent the mean error plus one standard deviation of the trichromat sample and sample with CVD.

**Figure 5:**
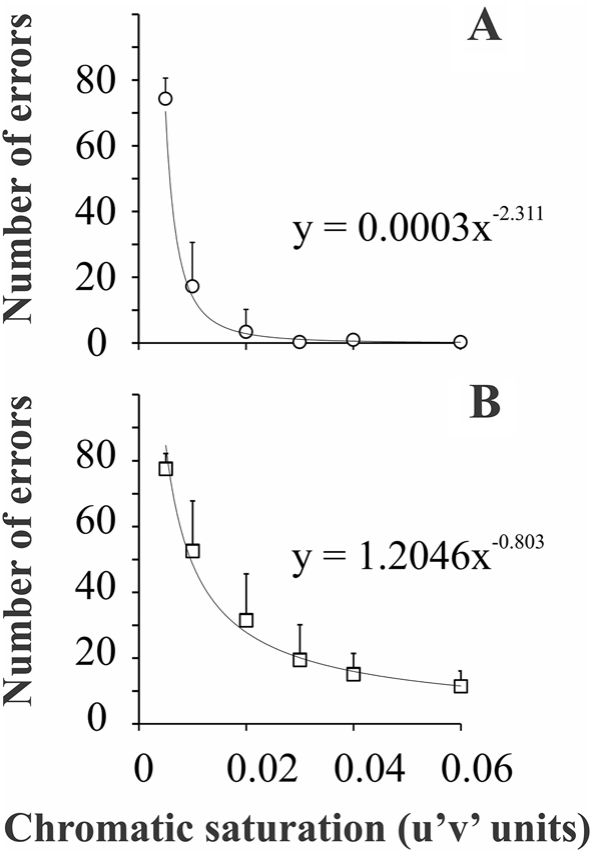
Mean performance of the chromatic discrimination as a function of the chromatic saturation of the target for normal trichromats **(A)** and participants with CVD **(B)**. Both groups increased the number of errors as the target was desaturated following a negative power trend (black curve) with higher exponent for normal trichromats than for participants with CVD. Circles and squares represent the mean number of errors from trichromats and participants with CVD, respectively. Error bars represent the standard deviation of the mean.

In general, we observed that at high chromatic saturation conditions, there were fewer errors. The number of errors of the sample increased as the chromatic saturation of the target decreased, following a negative power trend, as shown in Fig. 5. Both trichromats and participants with CVD showed the same general trend, with a higher exponent for normal trichromats (Fig. 5A) than for participants with CVD (panel Fig. 5B), due to the latter showing higher number of errors.

**Figure 6:**
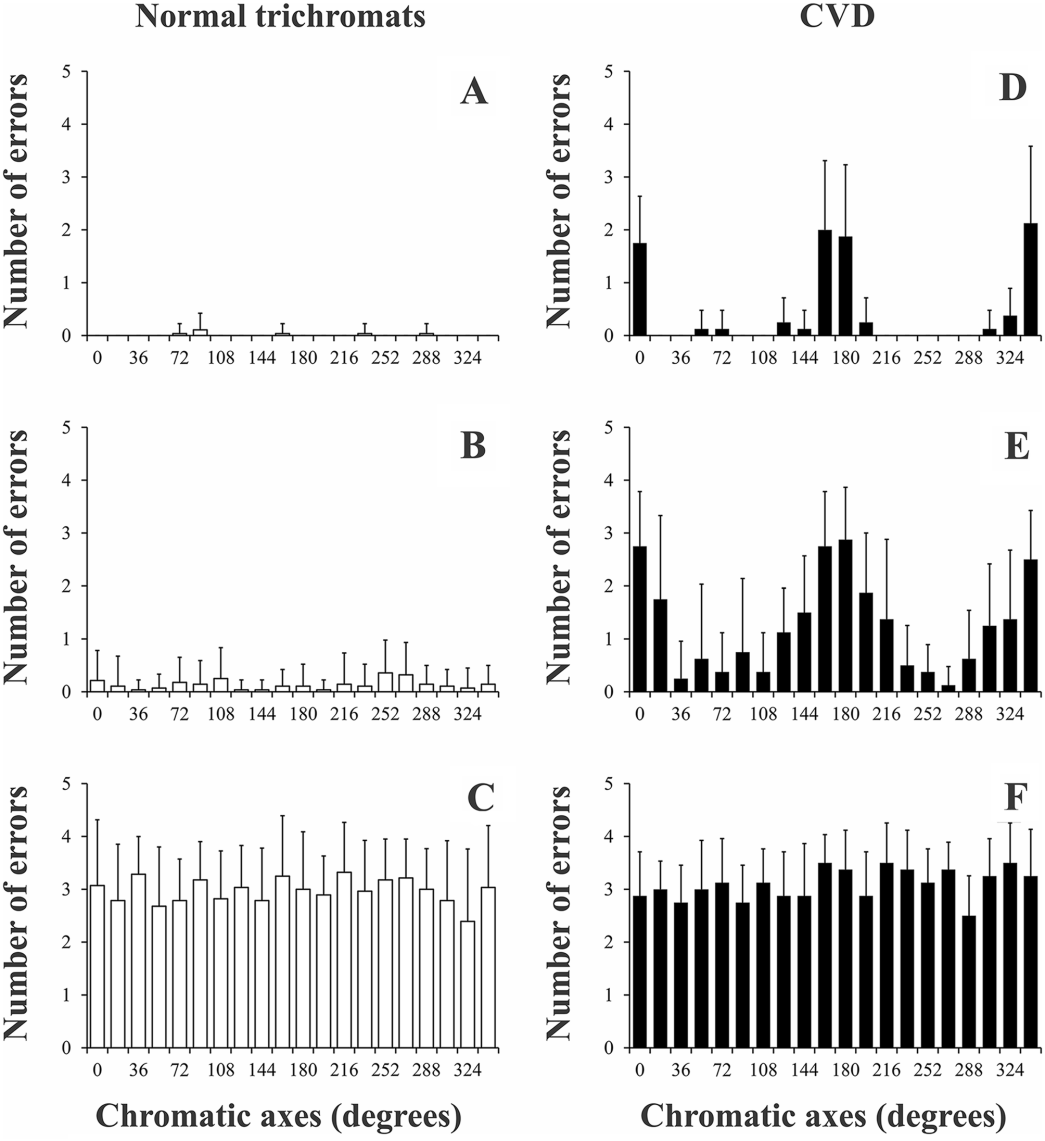
Mean performance of the chromatic discrimination for each chromatic vector in different stimulus saturation conditions for normal trichromats **(A–C)** and participants with CVD **(D–F)**. At high saturation condition, trichromats had none or few errors, while the participants with CVD failed mainly at the reg-green axes. At low saturation conditions both groups showed a similar pattern of errors.

Figure 6 shows the distribution of the errors along the chromatic vectors for trichromats and participants with CVD. The distribution of the errors of the trichromats across the chromatic vectors showed no systematic variation for saturation conditions of 0.06, 0.04, and 0.03 u’v’ units. However, from 0.02 to 0.005 u’v’ unit conditions, we observed a discrete prevalence of tritan errors (90°–108° and 252°–270° chromatic vectors), in agreement with slightly increased thresholds for the tritan vector in the CCT trivector protocol as previously published by others^12^. Then, it is reasonable that trichromats’ errors show first in chromatic axis close to the tritan color confusion line.

For participants with CVD, the distribution of the errors across the chromatic vectors occurred mainly in red-green chromatic vectors (0°–342° and 162°–180°) (Fig. 6D,E). As the chromatic saturation of the target decreases, the range of errors gradually extends to the neighbor vectors of the red-green vectors.

A three-way ANOVA with Geisser-Greenhouse corrections was conducted on the influence of three independent variables (color vision phenotype, chromatic saturation of the stimulus target, and chromatic vector). The results of the analysis of variance are shown in Table 2. We found that all factors (color vision phenotype, stimulus saturation, and chromatic vector) effects were statistically significant on the number of errors during the task, as well as their interactions. The ANOVA results were predicted because it was expected that (i) trichromats had better performance than participants with CVD (factor color vision phenotype), that (ii) in lower saturation conditions the number of errors were bigger (factor chromatic saturation) than in higher saturation values, and that (iii) in some chromatic vectors close to the color confusion lines (factor chromatic vector) we would found larger number of errors in the stimulus discrimination than in chromaticities out of the color confusion lines. The significance of the interactions among the factors indicated that the number of errors is influenced by the combination among the factors. For example, trichromats had a smaller rate of errors in high saturation levels and chromatic axes close to the color confusion lines than the participants with CVD in the same conditions of factors. The significance of the interactions is an indicator of great difference between both groups of data. The partial eta squared analysis showed that 5.42% of the variance was associated with color vision phenotype, 38.6% of the variance was associated with saturation level, and 3.58% of the variance was associated with the chromatic axes factor. This result leads to the conclusion that saturation was the most important main effect.

**Table 2:** Results of the three-way ANOVA on the number of errors during the perceptual task.

Variables	SS	DF	MS	F	p-value
Color vision phenotype (v1)	406.86	1	406.862	946.47	< 0.0001
Chromatic saturation (v2)	2900.08	5	580.015	1349.28	< 0.0001
Chromatic vector (v3)	269.43	19	14.181	32.99	< 0.0001
v1*v2	136.22	5	27.245	63.38	< 0.0001
v1*v3	339.02	19	17.843	41.51	< 0.0001
v2*v3	134.3	95	1.414	3.29	< 0.0001
v1*v2*v3	160.1	95	1.685	2.92	< 0.0001
Error	1757.31	4088	0.43		
Total	7506.36	4327			

*SS* sum of squares, *DF* degrees of freedom, *MS* mean square.

The results of the ROC analysis are shown in Table 3. The AUC was considered as the quantification for the performance to screen the presence of the CVD. We observed that saturations between 0.02 and 0.06 u’v’ units had accuracies close to 100% enabling a perfect screening of the participants with red-green CVD. The use of the saturation level of 0.01 and 0.005 u’v’ units decreased the performance of the classifier to screen the CVD. As we had small size of participants with CVD suggestive of protan (n = 2) and deutan (n = 8) defects, we did not calculate the accuracy for classification of the color vision deficiency.

**Table 3:** Results of the ROC analysis for the different stimulus saturation.

Stimulus saturation (u’v’ units)	Area under the curve (%)
0.06	100
0.04	100
0.03	99.78
0.02	100
0.01	90.09
0.005	81.9

## Discussion

The present study demonstrated that the proposed protocol for psychophysical chromatic discrimination at fixed saturation levels could distinguish the data obtained from subjects with normal trichromacy and people with congenital CVD. Furthermore, stimuli with saturation ranging between 0.06 and 0.02 u’v’ units showed high accuracy for the identification of CVD allowing their classification in either normal or deviant color vision.

Pseudoisochromatic stimuli have been used extensively in color vision tests because its design, including luminance and spatial noise, is very efficient in masking the chromaticity differences between the target and background^4^. Additionally, we have successfully used these stimuli designs to evaluate color vision changes in patients with genetic defects such as Duchenne Muscular Dystrophy^13^, Leber's Hereditary Optic Neuropathy^14,15^, and patients with acquired defects such as diabetes^16^, retinal toxicity by hydroxichloroquine^17^, and mercury vapor^18^ in studies that validated this methodology.

Many color vision tests use number of errors in the identification of the target in pseudoisochromatic plates such as the Ishihara and HRR plate tests, but those tests show several limitations: (1) They investigate the chromatic discrimination in few chromatic axes; (2) Since they are printed, the precision control of color and saturation is limited in short color ranges; (3) They are fixed in plates making it easy to memorize after several presentations; (4) The psychophysical task is a pass-fail procedure generating a nominal classification, and; (5) They require the identification of specific shapes, including letters, numbers, geometric forms, or pathways, which carry greater difficulty in execution compared to the C-gap orientation task used in the present study. Many investigations have reported the misreading of the Ishihara plates even in normal trichromats^19,20^, leading to a reduction of their sensitivity and reliability.

Shin et al.^8^ proposed a computerized approach to evaluate color vision using a test that quantified number of errors to identify targets of pseudoisochromatic stimulus in the screen. In their test, there were 80 pseudoisochromatic stimuli that combined different lightness, saturation and hues values. We understood that the use of the number of errors during the test could shorten the test duration, but variability across the stimulus conditions difficulted the characterization of the results or the association of the result with the characteristic of the stimulus. Then in the present study, we proposed to investigate the performance of fixed saturation level to evaluate congenital CVD.

We identified the phenotype of the participant’s color vision using a well-known commercial color vision test, CCT. In the participants who had suggestive color vision loss in the CCT, we evaluated the genetics of the L-cone and M-cone genes to confirm the hereditary background of the color vision loss. We understand that anomaloscopy is the gold-standard to classify CVD. As our sample size of participants with CVD is small and our purpose was not to classify the CVD in deutan or protan defects, but to identify the presence of CVD, this limitation did not interfere in the final results of the present investigation.

Although it is tempting to compare the test offered here with the variety of existing tests, the present study did not experimentally assess this issue. Our main contribution and novelty was to estimate the accuracy from different saturation levels to screen CVD. Our results add to the literature suggesting that the use of different stimulus conditions has potential to interfere in the accuracy of the test. For example, there are several reports about the efficiency of different plates in the Ishihara to evoke correct responses from trichromats or people with CVD^19^ and the different combination of saturation, lightness and hue present in the plates can potentially explain these differences in their efficiency in screening CVD. Eight participants with CVD were evaluated in the present study. We performed a ROC analysis to calculate the accuracy of the fixed saturation levels to identify the presence of red-green CVD. We observed that using saturation conditions equal and above of 0.02 u’v’ units, the accuracies were close 100%. Even using saturation of 0.005 or 0.01 u’v’ units, the accuracies were higher than 80%. Several studies have investigated the performance of other color vision tests to identify or classify the congenital color vision deficiencies. Data from 401 people with red-green CVD showed that the performance to identify the presence of CVD varied depending on the type of plates from Ishihara test or HRR test^19,21^. For Ishihara test, the combination of transformation and vanishing plates had 95% of accuracy to screen the CVD, while hidden digits plates had identified 50% of the participants with CVD. The protan/deutan classification plates had fails to classify 18% of the protans and 3% of the deutans. Similarly, HRR plates had screening accuracy of 100% for dichromats and 96.4% for anomalous trichromats. Protan/deutan classification was correct for 95% of the participants. As far as we know, there is no data of accuracy for CVD screening or classification of CCT ellipse and trivector protocols.

There is a trade-off relationship between number of chromatic axes to be evaluated and duration of the color vision test. The duration of the testing using CCT trivector protocol is similar to the duration of our proposal using 20 chromatic vectors. Considering purposes of diagnostic and classification of the CVD, the current proposal could be applied using only three vectors in the color confusion lines and the testing would be faster than the estimation of thresholds during CCT trivector protocols.

We are aware of some caveats and limitations to be overcome in future studies. The main limitation of the present study was the number of participants with CVD. Still, the results obtained from those eight participants were consistent, and we believe there is no qualitative loss of information or misinterpretation of the results shown here. Methodological studies regarding the computer-generated pseudoisochromatic stimuli reliability showed very consistent individual results, even if the procedure were replicated several times^22^.

In conclusion, we suggest that the accuracy of pseudoisochromatic stimulus in chromatic discrimination test to screen CVD can be influenced by the saturation levels present in the target of the stimulus. The combination of high and low saturation levels in the same color vision test can impair the accuracy of the test. Our results can contribute to the design of new color vision tests.

## Data Availability

The datasets generated during and/or analyzed during the current study are available from the corresponding author on reasonable request.
